# Combined Transcriptome and Proteome Analysis of the Protein Composition of the Brochosomes of the Leafhopper *Nephotettix cincticeps*

**DOI:** 10.3390/insects14100784

**Published:** 2023-09-26

**Authors:** Wei Wu, Zhuangxin Ye, Qianzhuo Mao, Hong-Wei Shan, Jun-Min Li, Jian-Ping Chen

**Affiliations:** State Key Laboratory for Managing Biotic and Chemical Threats to the Quality and Safety of Agro-Products, Key Laboratory of Biotechnology in Plant Protection of Ministry of Agriculture and Zhejiang Province, Institute of Plant Virology, Ningbo University, Ningbo 315211, China

**Keywords:** brochosome, Cicadellidae, RNA-Seq, LC–MS/MS, rice green leafhopper

## Abstract

**Simple Summary:**

The Cicadellidae family comprises over 22,000 described species, renowned for the presence of brochosome coatings on their integuments. Brochosomes are protein–lipid particles, hollow spheres with a honeycomb-like surface, synthesized and secreted by a specialized segment of leafhopper Malpighian tubules. Brochosomes are believed to assist leafhoppers in resisting various threats. However, the exact protein composition of brochosomes remains poorly understood. In this study, we conducted a combined transcriptome and proteome analysis of the protein composition of brochosomes in the leafhopper *Nephotettix cincticeps*. Ultimately, we identified 22 candidate brochosome proteins. These proteins were divided into two groups: brochosomins (BSM) and brochosome-associated proteins (BSAP). Examination of conserved motifs and functional predictions unveiled potential roles for these proteins. Our findings indicated that BSM, along with some BSAP, are exclusive to the Cicadellidae family of leafhoppers. These findings provide insights into brochosome synthesis, function, and evolutionary origins in leafhoppers, highlighting the existence of species-specific orphan genes encoding BSM and some BSAP. Furthermore, this research underscores the complexity of brochosome assembly and its potential roles in leafhopper biology.

**Abstract:**

Brochosomes, unique coatings on the integuments of Cicadellidae, are synthesized in specialized glandular sections of Malpighian tubules. However, limited knowledge exists regarding the protein composition of brochosomes. In this study, we conducted transcriptomic and proteomic profiling to characterize the brochosome protein composition in the rice green leafhopper *Nephotettix cincticeps*. Brochosomes were collected from the forewings of leafhoppers using ultrasonic treatment, allowing for more effective brochosome collection and shaking treatment, resulting in purer brochosomes. Transcriptome sequencing analysis identified 106 genes specifically expressed in the Malpighian tubules; combined with proteomic data, we identified 22 candidate brochosome proteins. These proteins were classified into 12 brochosomins (BSM) and 10 brochosome-associated proteins (BSAP) based on previous research. Conserved motif analysis and functional predictions unveiled unique motifs in each BSM, while BSAP appeared to play a crucial role in BSM folding and pathogen resistance. Comparative analysis of other Hemiptera species demonstrated that all BSM and some BSAP are specific to the Cicadellidae family. Our findings could contribute to understanding the mechanism of brochosome synthesis, its function, and evolutionary genesis.

## 1. Introduction

The Cicadellidae insect family is the largest in the Hemiptera order, comprising 25 subfamilies with over 22,000 described species. Brochosome coatings are a common feature found in all subfamilies and major tribes of Cicadellidae [1,2]. Brochosomes exhibit varying morphologies across leafhopper species, typically appearing as hollow spheres with diameters ranging from 0.2–0.6 µm and possessing a honeycomb-like surface [3]. Brochosomes are synthesized in the Malpighian tubules, extruded through the hindgut after molting and subsequently applied as a coating onto the new integument [1,2,4]. The function of brochosome in leafhoppers is uncertain despite several hypotheses. It is thought to act as a protective epidermal coating, safeguarding against excrement, water, pathogens, and predators [1,2,5,6]. However, empirical evidence is lacking for most of its functions, despite its hydrophobicity and its potential to assist leafhoppers in evading spider webs and blocking parasitic eggs [5,6,7].

Brochosomes are synthesized in the distal segment of the leafhopper’s Malpighian tubules [1,2,4]. In most insects, the Malpighian tubules function as major excretory organs responsible for primary urine production and osmotic pressure regulation [8,9]. Moreover, certain insects showcase distinct roles of Malpighian tubules. These roles encompass the generation, modification, and retention of mucopolysaccharides, proteins, fibers resembling silk, as well as mucofibrils [9,10,11,12,13]. These specialized functions are associated with modifications of cells within the entire tubules, specific segments, or occasionally modified secretory cells dispersed throughout the Malpighian tubules [9]. In Cicadellidae, the Malpighian tubule is divided into proximal, distal, and terminal segments [14,15]. The distal segment, responsible for brochosome synthesis, is characterized by its rod-shaped, inflated, and thick structure [3]. The lumen of the distal segment is surrounded by a single layer of secretory cells with large, spherical nuclei and an extended, rough endoplasmic reticulum [14,15]. Within Golgi-derived vacuoles, brochosomes acquire their distinctive honeycombed surface through the shaping of initially spherical particles with closely-set invaginations [3,4]. Early histochemical and enzymological tests demonstrated that the brochosome is a particle generated by a protein–lipid complex [16,17]. Comprehensive CHN elemental and amino acid studies of brochosomes from two leafhopper species (*Paraphlepsius irroratus* and *Graphocephala fennahi*) have indicated that brochosomes are primarily composed of glycine-, tyrosine-, and proline-rich proteins, with proteins accounting for 45–75% of their composition [2,18]. Recent studies on leafhoppers *G. fennahi* and *Homalodisca vitripennis* have identified the brochosome as predominantly composed of brochosomins (BSM) and brochosome-associated proteins (BSAP) [18,19]. BSM, a novel family of secreted proteins ranging from 21–40 kDa, serve as the major structural component of brochosomes [18]. The BSAP category includes proteins with tandem poly-proline helices, cyclase-like BSAP, glycine-, tyrosine-, proline-rich BSAP (G, Y, P-rich BSAP), and other BSAP [18,19]. Moreover, except for several minor BSAP possibly related to cyclases, BSM and BSAP have only been identified in the five families of the Membracoidea superfamily (Cicadellidae, Myerslopiidae, Atalionidae, Membracidae, and Melizidae), but not in other Hemiptera species. This suggests that these proteins are encoded by taxonomically restricted gene families, also known as orphan genes [18].

The rice green leafhopper *Nephotettix cincticeps* (Hemiptera: Cicadomorpha: Membracoidea: Cicadellidae), one of the most destructive insect pests on rice, is widely distributed in all rice-growing areas in China and east Asian countries. It causes damage through direct feeding and by transmitting virus and phytoplasma pathogens, such as rice dwarf virus and rice yellow dwarf phytoplasma [20,21,22]. The integuments of *N. cincticeps* are covered with brochosomes; however, the protein composition of these brochosomes remains unknown. We conducted transcriptome sequencing analysis on different leafhopper tissues to identify genes specifically expressed in Malpighian tubules. Two methods for isolating and extracting brochosomes were compared, and their identification was performed using LC–MS/MS. After combined proteomic and transcriptomic analyses, we identified 22 candidate brochosome proteins and analyzed their classification, conserved domains, motifs, and functions. Our results offer valuable insights into brochosome synthesis, function, and evolutionary origins.

## 2. Materials and Methods

### 2.1. Insects

The rice green leafhopper *N. cincticeps* adults were collected from a rice field in Jiaxing, Zhejiang Province, China, and maintained in insect-proof greenhouses at 26 ± 1 °C under a 16:8 h light:dark photoperiod and 50 ± 5% relative humidity on rice variety TaiChung Native 1 (TN1).

### 2.2. Sample Preparation and Illumina Sequencing

The adult *N. cincticeps* were frozen, anesthetized on ice, and their salivary gland (Sg), midgut (Mg), Malpighian tube (Mt), and residual body (Rb) were dissected on ice-cold sterile phosphate buffer solution (PBS, pH = 7.2) treated with 0.1% diethylpyrocarbonate under a stereomicroscope. Total RNA from each tissue was prepared with a Trizol Reagent Kit (Invitrogen, Carlsbad, CA, USA), according to the manufacturer’s protocol. The degradation and contamination of total RNA were analyzed with Bioanalyzer 2100 and RNA 1000 Nano LabChip Kit (Agilent, Palo Alto, CA, USA). After extracting total RNA, mRNA was purified from total RNA (5 μg) using poly-T oligo-attached magnetic beads. The isolated mRNA was fragmented using a fragmentation buffer. These fragments were then used as templates for reverse transcription, resulting in the final cDNA library. The mRNASeq sample preparation kit protocol (Illumina, San Diego, CA, USA) was followed. Subsequently, paired-end sequencing was conducted on an Illumina Hiseq 4000 at LC Sciences, USA, following the vendor’s protocol.

### 2.3. Transcriptomic Analysis

The raw data from high-throughput sequencing underwent quality filtering. This involved using Trimmomatic (version 0.36) to obtain high-quality clean reads, which entailed removing adapters, low-quality sequences, and N bases. The clean reads were then mapped to the reference genome using HISAT2 [23]. Gene expression values were quantified in transcripts per million (TPM) using StringTie [24]. Differential expression analysis involved comparing the TPM values of genes in Mt with the other three tissues. Gene differential expression analysis was performed using the R package edgeR [25]. Mt-specific genes were identified based on a TPM value > 100 in Mt and at least one set matching the criteria of the fold change ratio > 4 and *p*-value < 0.001 in MT vs. the other three samples. Signal peptides were identified using SignalP 5.0 Server (https://services.healthtech.dtu.dk/services/SignalP-5.0/, accessed on: 25 October 2022), and transmembrane helices in proteins were predicted using THMHH Server v. 2.0 (https://services.healthtech.dtu.dk/services/TMHMM-2.0/, accessed on: 25 October 2022).

### 2.4. Tissue-Specific Expressions Analysis

The insect tissue from Sg, Mg, Mt, and Rb were dissected under the stereomicroscope. Total RNA was extracted from each tissue sample using Trizol reagent (Invitrogen, Carlsbad, CA, USA) following the protocol. Genomic DNA contamination removal and cDNA synthesis were carried out using the NovoScript Plus All-in-one 1st Strand cDNA Synthesis SuperMix Kit (Novoprotein, Suzhou, Jiangsu, China). Quantitative PCR (qPCR) was performed using the QuantStudio 5 Real-Time PCR System (ThermoFisher, Waltham, MA, USA) and the NovoStart SYBR qPCR SuperMix Plus Kit (Novoprotein, Suzhou, Jiangsu, China). The first-strand cDNA and a no-reverse-transcription control were employed as templates for four independent biological replicates, following this thermal cycling program: an initial denaturation step at 95 °C for 5 min, followed by 40 cycles of 95 °C for 15 s, 60 °C for 30 s, then 95 °C for 15 s, and 60 °C for 1 min, concluding with a melt curve ranging from 60 °C to 95 °C. Gene-specific primers were designed using the Primer Premier 6.0 software. The relative gene expression levels were normalized to the expression of EF1α and determined using the 2^−ΔΔ^Ct (cycle threshold) method. The names and sequences of the gene-specific primers can be found in Table S1.

### 2.5. Brochosomes Collection

To detect the protein composition of the brochosomes, we collected brochosomes from leafhoppers using two methods. According to a previous report [18], forewings dissected from 2000 leafhoppers were placed in a 50 mL centrifuge tube filled with high grade acetone. After 15 min, the tube was sonicated for 2 min to facilitate separation of BS from the forewings. To remove coarse debris, the acetone was decanted and filtered by gravity through filter paper. The brochosome was precipitated from the filtrate by centrifugation at 1000× *g* for 10 min. The supernatant was carefully removed and replaced with fresh acetone. The particles were resuspended in fresh acetone with a brief ultrasonic treatment and then centrifuged again at 1000× *g* for 10 min; this procedure was repeated three times. BS-1 is the name of the final brochosome sample collected. In addition, we used another method to facilitate the separation of BS from the forewings. Forewings dissected from 2000 leafhoppers were placed in a 50 mL centrifuge tube filled with high grade acetone. The centrifuge tube was placed on a roller vibrator at room temperature at 50 rpm for 12 h. During rotation, the brochosomes are separated from the forewings by the mutual friction of the forewings in acetone. The brochosome was precipitated from the acetone by centrifugation at 1000× *g* for 10 min. The particles were resuspended in fresh acetone with a brief ultrasonic treatment and then centrifuged again at 1000× *g* for 10 min; this procedure was repeated three times. BS-2 is the name of the final protein sample collected. These two samples independently underwent protein digestion and nano-LC–MS/MS analysis.

### 2.6. Electrophoresis

To compare proteins in BS-1 and BS-2, SDS-PAGE gel electrophoresis was employed. Dry pelleted brochosomes were directly resuspended in 100 μL of 1× SDS-PAGE sample buffer, heated to 100 °C for 5 min, and then centrifuged. Subsequently, 10 μL of each prepared sample was loaded into the respective wells of the gel. The stacking gel consisted of 4% acrylamide, while the resolving gel was composed of 10% acrylamide. Electrophoresis was conducted at 25 mA per gel for a duration exceeding 1.5 h. Post-electrophoresis, the gels were stained using the eStain L1 Protein Staining System from GenScript and imaged. A color-prestained protein standard (Genstar, San Francisco, CA, USA, 10 to 245 kDa) was utilized as a marker for protein size.

### 2.7. Transmission Electron Microscopy

To perform a detailed morphological analysis of brochosomes in BS-1 and BS-2, specimens underwent transmission electron microscopy (TEM) using a conventional negative staining procedure. Brochosome pellets were suspended in 20 µL of PBS, then applied to 200-mesh copper grids coated with formvar film and left for 2 min. After a gentle wash with filtered PBS, the brochosome pellets on the grids were fixed immediately with 2.5% glutaraldehyde for 1 min. Following this, the grids were exposed to a 2% (*w*/*v*) sodium phosphotungstate solution for 1 min, and any excess liquid was removed using filter paper. Subsequently, the negatively stained specimens were observed using a Hitachi electron microscope model HT7800 (Hitachi, Japan).

### 2.8. Protein Identification by LC–MS/MS

Brochosomes were subjected to digestion using the FASP method, following a previously established procedure. In summary, BS-1 and BS-2 were washed twice with 25 mM ammonium bicarbonate (ABC), and disulfide bonds were reduced using 10 mM Tris 2-carboxyethyl phosphine (TCEP) at 37 °C for 1 h. The resulting thiols were then alkylated with 12 mM iodoacetamide for 20 min, followed by centrifugation to remove excess liquid. The proteins were washed twice with 100 μL of 50 mM ABC. Digestion was carried out by adding 1 μg of trypsin (Promega sequencing grade) in 200 μL of 50 mM ABC, followed by overnight incubation at 37 °C. The resulting digested peptides were collected through centrifugation at 14,000× *g* for 10 min and quantified based on their optical density (OD) 280 values.

Subsequently, the peptides were separated using an Agilent 1260 Infinity II HPLC system (Thermo Scientific) equipped with a C18 trap column and analyzed using a Q Exactive mass spectrometry system (Thermo Scientific). The C18 column was prepared with a linear solvent gradient using solvent A (0.1% formic acid in water) and solvent B (100% acetonitrile with 0.1% formic acid) for column washing. Peptides were loaded onto the C18 column and eluted using the following gradient: 0–5 min, 6% B; 5–75 min, 6–38% B; 75–85 min, 38–100% B; 85–90 min, 100% B, all at a flow rate of 300 nL min^−1^.

During mass spectrometry analysis, the Q Exactive system operated in an information-dependent mode, alternating between full-scan MS and MS/MS acquisition. Mass spectra were acquired within the range of 350 to 1800 *m*/*z*, maintaining a resolution of 70,000 and a maximum injection time of 50 ms per spectrum.

### 2.9. Bioinformatics Analysis

Protein identification was conducted using MaxQuant software [26]. MS/MS spectra were searched against a query database with the MaxQuant search engine. This database was predicted based on the leafhopper *N. cincticeps* genome database [27]. Retrieval parameters were set as follows: trypsin/P for digestion, allowing for up to two missed digestion sites, and requiring a minimum peptide segment length of seven amino acids. Up to five modifications were allowed per peptide segment. The primary parent ion mass error tolerance for the first search was set to 20 ppm, and for the main search, it was set to 5 ppm. The mass error tolerance was 0.02 Da. Cysteine alkylation was fixed, while methionine oxidation was considered a variable modification. TMT-6plex was selected as the quantitative method, and the false discovery rate for both protein and PSM identification was set at 1%. The common proteins identified in BS-1 and BS-2 were determined using Venn diagram analysis through TBtools (version 1.098769) [28].

### 2.10. Distribution of Brochosome Candidate Proteins in Leafhopper and Hemiptera

The brochosome is an enigmatic secretion produced by leafhoppers (Cicadellidae). To determine the distribution of brochosome candidate proteins in leafhoppers and other Hemiptera insects, the Malpighian tube-specific protein-coding genes were identified based on a TPM value > 100 in the Malpighian tube, the criteria of the fold change ratio > 4 and *p*-value < 0.001 in the transcriptome. Only Malpighian tube-specific proteins identified in BS-1 and BS-2 will be considered as brochosome candidate proteins. Conserved domains and motifs in the brochosome candidate proteins were discovered by NCBI Conserved Domains (http://www.ncbi.nlm.nih.gov/Structure/cdd/wrpsb.cgi, accessed on: 5 November 2022) and MEME (http://meme-suite.org/tools/meme, accessed on: 5 November 2022).

The brochosome candidate proteins were used to search for homologous sequences in leafhopper transcriptomes or hemipteran genomes. Transcriptome data of 44 species of leafhopper were downloaded from the NCBI Sequence Read Archive, and reads were assembled with Trinity (version 2.4) [29]. Genomes of 45 species of Hemiptera insects were downloaded from the InsectBase 2.0 [30]. BLAST alignment with an E-value of 1e–5, a bit-score of 200, and a percent identity of 40% as thresholds were used to find brochosome candidate proteins homologous sequences of queries from the leafhopper transcriptomes or Hemiptera insect genome.

## 3. Results

### 3.1. Nephotettix Cincticeps Transcriptome Sequencing

**Figure 1:**
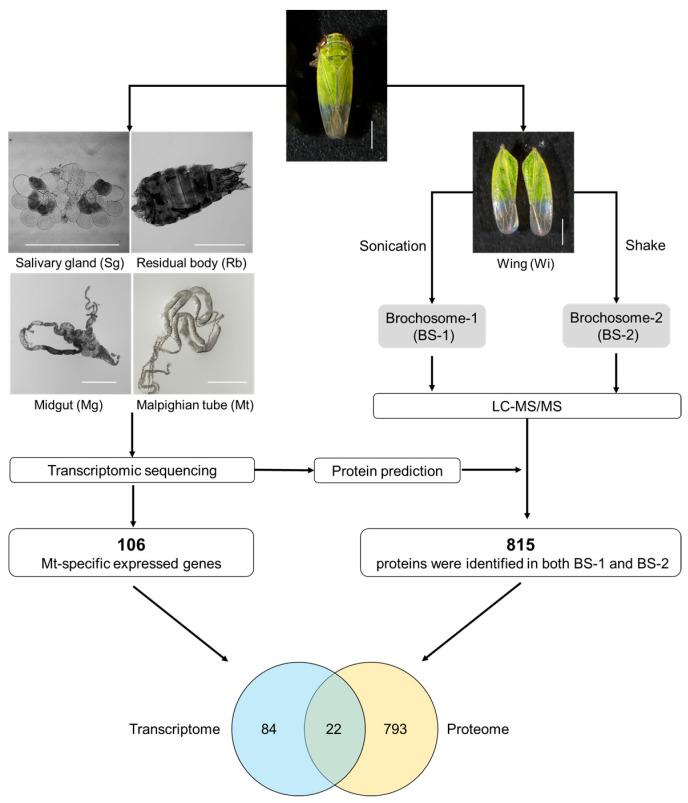
Workflow for the identification of brochosome candidate protein.

As illustrated in Figure 1, our workflow for identifying rice green leafhopper *N. cincticeps* brochosome candidate proteins based on shotgun proteomics data included three steps: transcriptome identification, peptide identification, and candidate protein screening.

**Figure 2:**
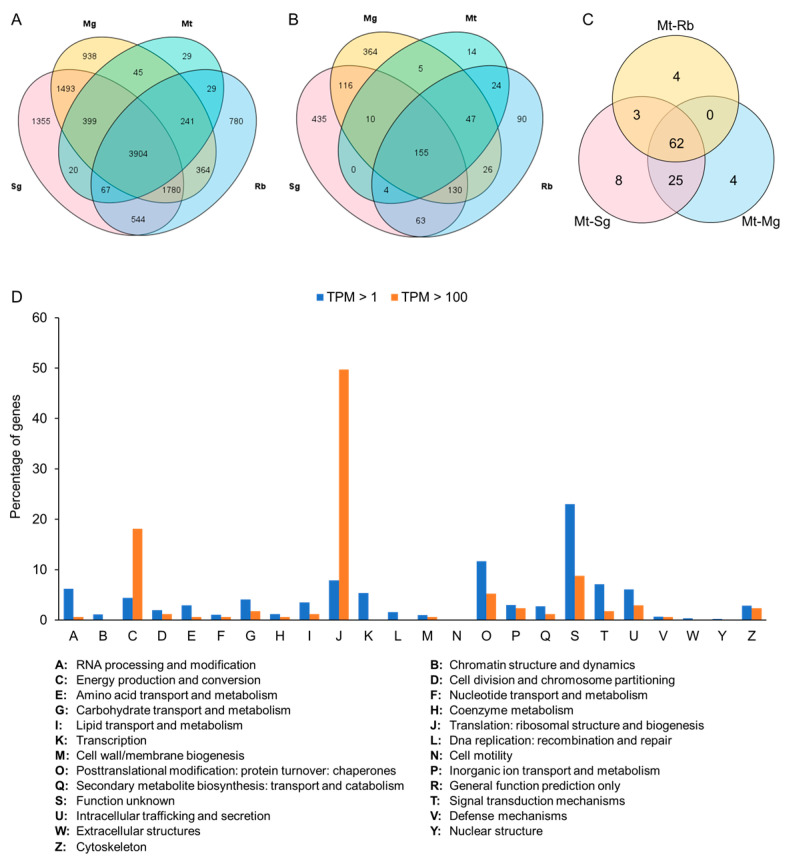
Screening for Mt-specific expression genes. (**A**,**B**) Venn diagram of genes in Sg, Mg, Mt, and Rb of leafhopper *N. cincticeps* with transcript levels of TPM > 1 (**A**) and TPM > 100 (**B**). (**C**) Venn diagrams were constructed based on a TPM value > 100 in Mt, a fold change ratio > 4 and a *p*-value < 0.001 for the three pair-wise comparisons (Mt vs. Sg, Mt vs. Mg, and Mt vs. Rb). (**D**) COG functional classification of gene transcript levels TPM > 1 and TPM > 100 in Mt.

Transcriptome sequencing was performed on the Sg, Mg, Mt, and Rb of *N. cincticeps*. After quality filtering, 43.21, 53.88, 55.17, and 45.55 million clean reads were obtained from Sg, Mg, Mt and Rb, respectively (Table S2). In total, 11,988 genes were identified to be expressed in at least one sample with transcripts per million mapped transcripts (TPM) > 1, of which there were 9562 genes in Sg, 9164 genes in Mg, 4734 genes in Mt, and 7709 genes in Rb (Figure 2A, Table S3).

The Sg, Mg, Mt, and Rb of the leafhopper were subjected to transcriptome sequencing analysis. Transcriptomes and their expression levels were determined by aligning the sequencing data to the *N. cincticeps* genome reference. Malpighian tubule-specific expressed genes were screened. Brochosome candidate proteins were collected from the forewings of *N. cincticeps* by ultrasonication or oscillation in a centrifuge tube containing acetone, followed by liquid chromatography-tandem mass spectrometry analysis. Protein identification was performed using the MaxQuant software and a query database generated from the *N. cincticeps* genome. Scale bar = 1 mm.

### 3.2. Screening of Mt-Specific Expression Genes

Previous studies have indicated that brochosomes are produced in the Mt of leafhoppers [3,4]. To screen for possible brochosome protein synthesis genes, we classified the Mt-expressed genes into three groups based on their expression levels. Most of the genes (n = 3617) were classified as low expression titers with transcript level TPM < 10. The remaining genes were classified as medium (10–100 TPM) and high (TPM > 100) expression rates, with 858 and 259 genes (Figure 2B, Table S3), respectively. Analysis of the Cluster of Orthologous Groups (COG) database revealed that the highly expressed genes in Mt were enriched in the C category “energy production and conversion” (31, 18.13%) and the J category “translation, ribosomal structure and biogenesis” (85, 49.70%) (Figure 2D and Figure S1). In total, 106 Mt-specific expression genes were identified in at least one of the three pair-wise comparisons, with 62 genes found in all three comparisons (Figure 2C). Most of the specific genes in Mt were annotated as uncharacterized proteins (Table S4).

The brochosome gradually matures inside Golgi-derived vesicles of specialized cells comprising glandular segments of the Malpighian tubules of leafhoppers. In the classical protein secretion pathway, an N-terminal signal peptide is found in proteins secreted via the endoplasmic reticulum–Golgi pathway [31]. Among 106 Mt-specific expressed genes, 71 proteins had a putative secretory peptide and no transmembrane domain or the only transmembrane domain was in the range of the signal peptide (Table S4), indicating the possible secretory capability of proteins.

To further validate the RNA-seq results, we selected 20 genes from the pool of 106 Malpighian tube-specifically expressed genes for RT-qPCR validation. All 20 genes showed significantly higher expression levels in the Malpighian tube compared to other tissues, with particular prominence observed in 00008770-RA, 00008771-RA, 00010774-RA, 00011528-RA, and 00012525-RA, which exhibited expression levels in the Malpighian tube over 100-fold higher than in other tissues (Figure S2). The RT-qPCR results were largely consistent with the RNA-seq data, providing further support for the reliability of the transcriptome sequencing data.

### 3.3. Morphology and Protein Composition Analysis of Brochosome

**Figure 3:**
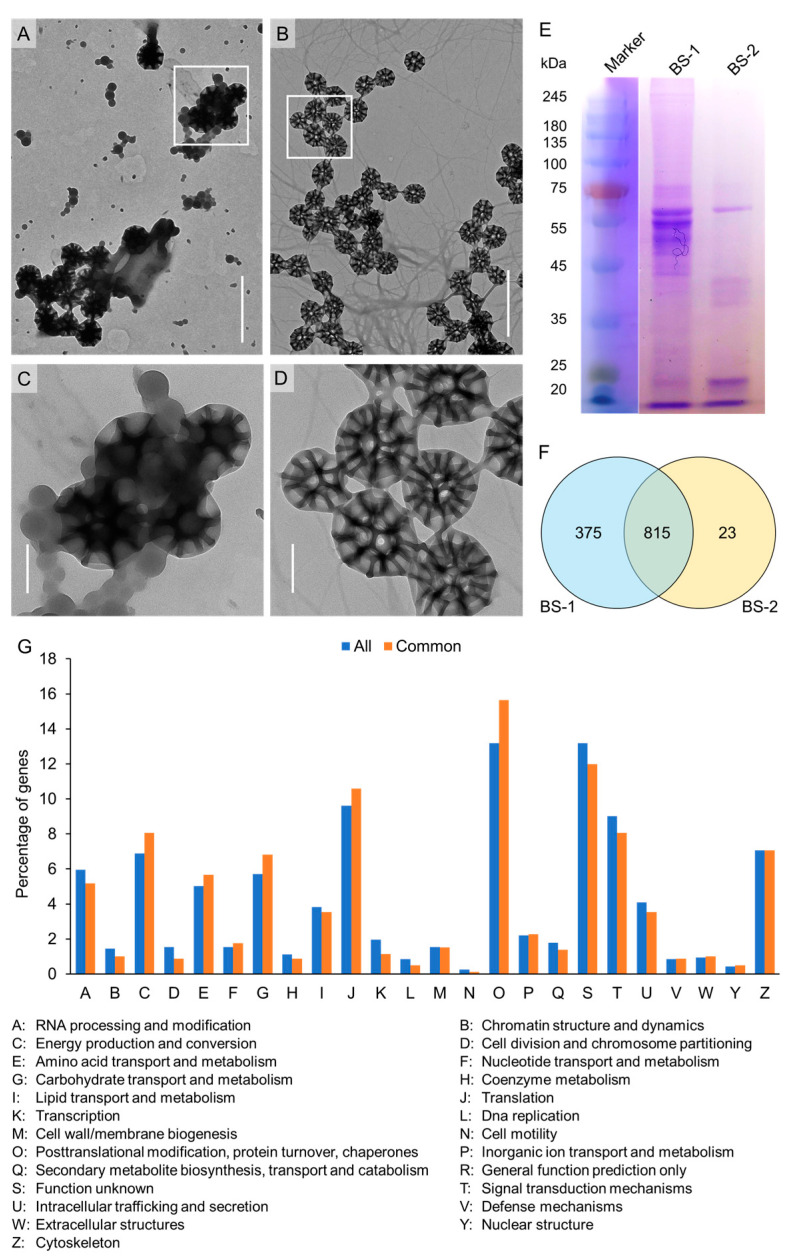
Identification of protein composition in *N. cincticeps* brochosome. (**A**–**D**) The morphology of brochosomes in BS-1 (**A**,**C**) and BS-2 (**B**,**D**) were observed under electron microscopy. Panels C and D are enlarged boxed areas on panels (**A**,**B**). Scale bars in A and B: 1 μm; scale bars in **C** and **D** 200 nm. (**E**) Proteins from brochosome of *N. cincticeps* resolved on 10% SDS-PAGE gel. (**F**) Venn diagrams of identified proteins in BS-1 and BS-2. (**G**) COG functional classification of all identified and common proteins in BS-1 and BS-2.

The brochosome (BS) collected by sonication and shaking under an electron microscope displayed a diameter of 350 nm with a honeycombed outer wall and a near-spherical central cavity. This indicated that the BS could be washed off the forewings with acetone, centrifuged, and dried to maintain normal morphology (Figure 3A–D), as previously reported [18]. Many impurities were observed around the BS in the BS-1 sample but not in the BS-2 sample (Figure 3A–D). The results of SDS-PAGE gel electrophoresis were consistent with the electron microscope findings. In the lane of the BS-2 sample, five main protein bands were observed in the range of 15–75 kDa, while the BS-1 sample showed numerous protein bands in the range of 40–75 kDa (Figure 3E and Figure S3).

### 3.4. Proteins Identified by LC–MS/MS

LC–MS/MS analysis identified 4224 peptides that matched 1195 proteins (Tables S5 and S6). BS-1 and BS-2 samples exposed 1172 and 838 proteins, respectively, with 815 proteins presented in both (Figure 3F, Table S6). Among all identified proteins, cuticle protein (00008794-RA) exhibited the highest peptide coverage (85.3%). In particular, in the BS-1 sample, six of the top ten proteins in terms of peptide coverage were cuticle proteins. Cuticle proteins (52, 4.35%) were the second most abundant proteins identified after ribosomal proteins (61, 5.20%). A total of 230 proteins were found to contain signal peptides. Using the COG database, 1113 proteins (94.96%) were mapped and grouped into 24 COG categories. The five largest categories were the O category, “posttranslational modification, protein turnover, chaperones” (155, 13.93%), the S category “function unknown” (155, 13.93%), the J category “translation, ribosomal structure and biogenesis” (113, 10.15%), the T category “signal transduction mechanisms” (106, 9.02%), and the Z category “cytoskeleton” (83, 7.46%) (Figure 3G).

### 3.5. Brochosome Candidate Proteins Screening

We combined the transcriptome and LC–MS/MS data to assess potential brochosome component proteins and identified 22 Malpighian tube-specific proteins in the proteome (Figure 1). Among these 22 proteins, the smallest and largest protein molecular weights were 13.2 and 75.8 kDa, respectively, consistent with the distribution of the brochosome on SDS-PAGE gels (Table S6). Most of these 22 proteins were annotated as uncharacterized proteins (18/22) in the *N. cincticeps* genome.

**Figure 4:**
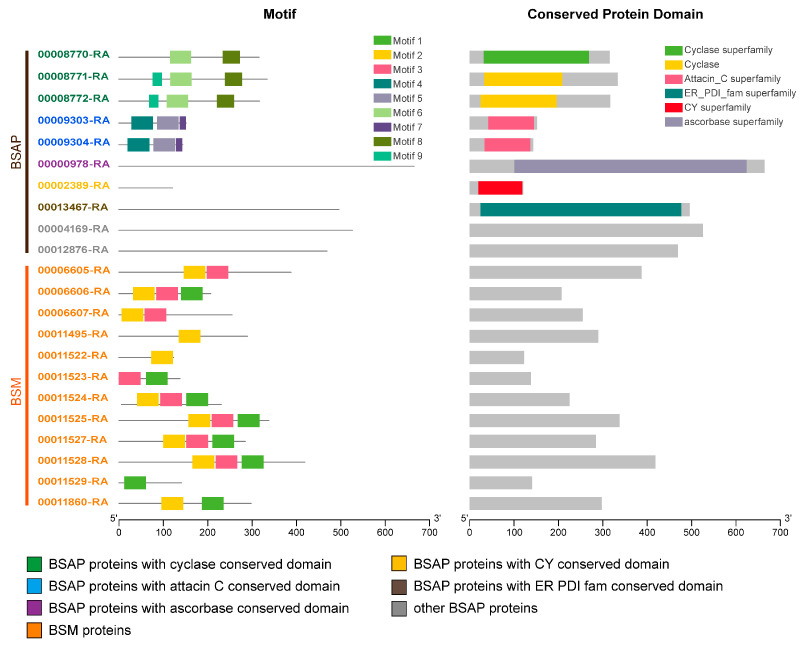
Analysis of motifs and conserved domains in brochosome candidate proteins. TBtools was employed to visualize motifs. Motifs were analyzed using the MEME online tool (**left**), and the protein domains analysis was performed using the NCBI-CDD database (**right**). Different colored blocks were used to represent each motif and conserved domain. The position and length of each colored box represent the actual motif and conserved domain size.

Based on prior studies, the brochosome protein fraction from two leafhopper species, *G. fennahi* and *H. vitripennis*, has been identified and categorized as BSM and BSAP [18,19]. We divided the 22 proteins into two groups based on functional annotation, conserved domain/motif, and homology with the leafhopper *G. fennahi* and *H. vitripennis* brochosome protein [18,19]. The first group consisted of 12 proteins containing one or more of the three conserved motifs and were homologous to BSM in *G. fennahi*. Intercomparison of all 22 protein sequences revealed partial similarity among these 12 protein sequences that contained the same conserved motif, suggesting that they are paralogous homologs (Figure 4 and Figure S4). Therefore, we hypothesized that these 12 proteins are the primary components of the *N. cincticeps* brochosome (BSM). The other group, BSAP, comprised 10 proteins. Eight of these proteins had conserved structural domains: three had a cyclase conserved domain (00008770-RA, 00008771-RA, and 00008772-RA), two had an attacin C conserved domain (00009303-RA and 00009304-RA), and the remaining three had conserved domains of the ER PDI fam (00013467-RA), CY (00002389-RA), and ascorbase (00000978-RA), respectively (Figure 4 and Figure S4).

### 3.6. Taxonomic Distribution of Brochosome-Related Proteins

**Figure 5:**
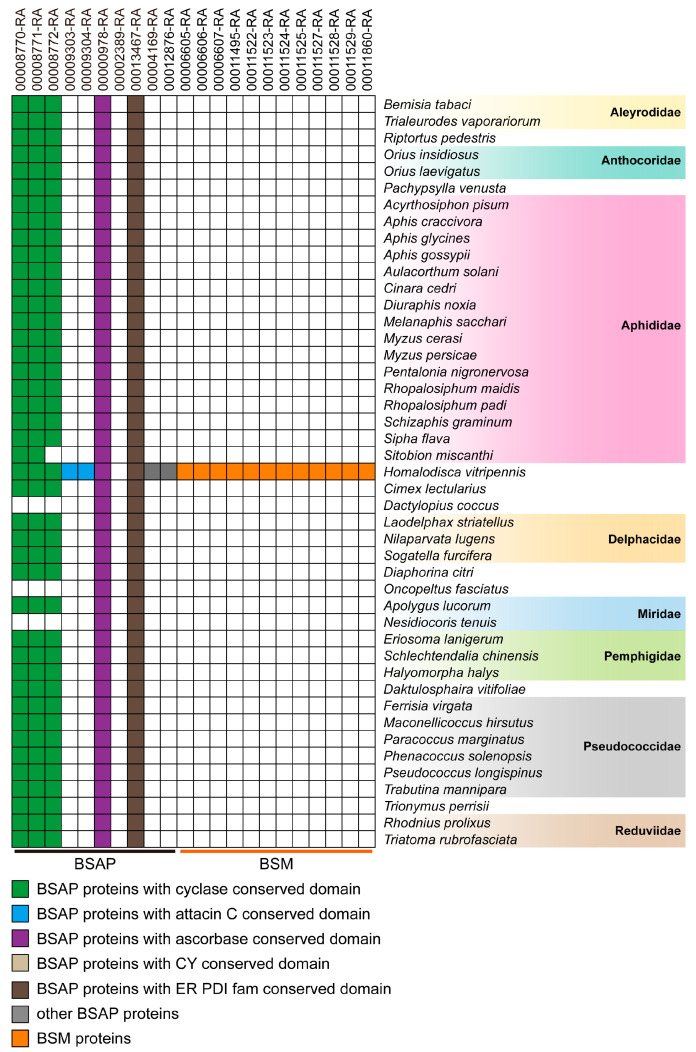
Identification of brochosome candidate proteins in different hemipteran species. The white color represents the absence of the homologous gene, and the color indicates its presence.

**Figure 6:**
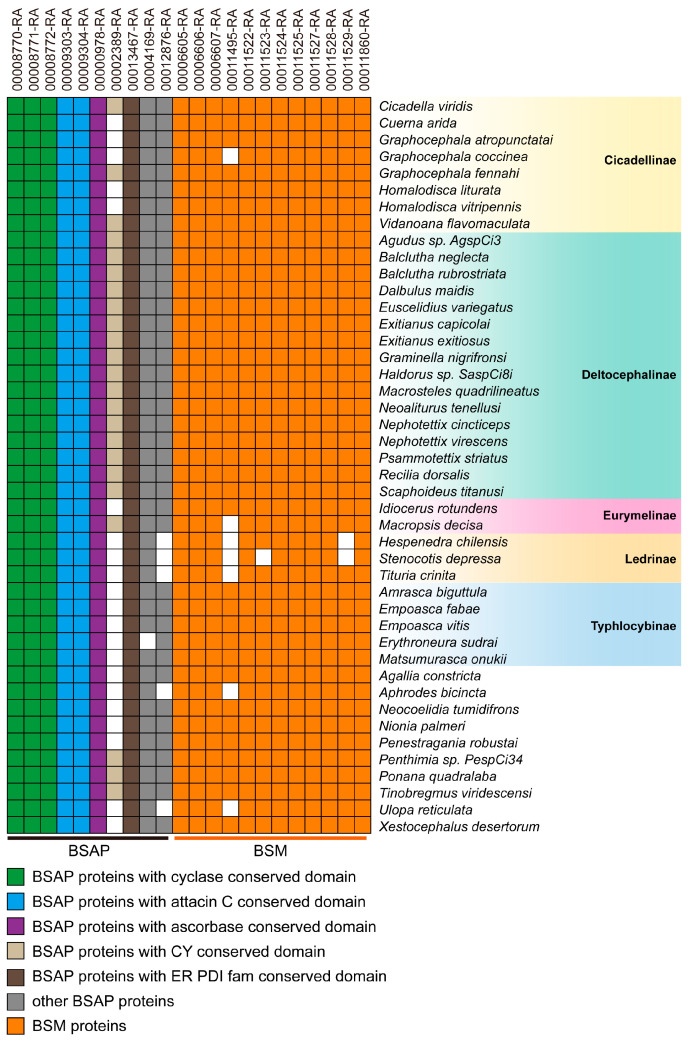
Identification of brochosome candidate proteins in different leafhopper species. The white color represents the absence of the homologous gene, and the color indicates its presence.

Brochosome is a specific type of secretion synthesized by the Malpighian tubes and is exclusive to members of the Cicadellidae (leafhoppers) family [2,3,18]. To determine whether the putative brochosome-related proteins were unique to the leafhopper family, these proteins were used to search against 45 currently available hemipteran genomes. As a result, five proteins showed homology in most hemipterans. Three of these proteins were homologous to BSAP in *G. fennahi*, which belongs to the cyclase superfamily [18]. Additionally, we found 21 protein homologs in the glassy-winged leafhopper *H. vitripennis* and 12 proteins homologous to the BSM of *G. fennahi* (Figure 5). Furthermore, when comparing these proteins to 44 other leafhopper species, it was found that almost all proteins were homologous to other leafhopper species (Figure 6). Based on the above results, it can be deduced that the brochosome BSM has no orthologues homologs in other Hemiptera species except Cicadellidae, which suggests that all BSM and some BSAP may be encoded by a class of genes specific to Cicadellidae.

## 4. Discussion

Brochosome is a distinctive coating on the integuments of leafhoppers [1,2,5,6]. In this study, we employed transcriptomics to characterize different tissues of *N. cincticeps* and identified putative Malpighian tube-specific expressed genes through strict screening conditions. Additionally, proteomics analysis was conducted to examine the protein composition of brochosomes obtained using sonication and shaking procedures. We successfully identified 1195 proteins, 815 of which were present in both samples. From these results, we identified 22 putative brochosome-related proteins, including 12 putative BSM proteins. Notably, homologs of the BSM were exclusive to the Cicadellidae family and absent in other Hemiptera species, indicating their potential encoding by a class of genes specific to Cicadellidae.

Brochosomes are produced in specialized glandular segments of the Malpighian tubules [3,4]. Malpighian tubules, which function as the primary excretory organs in most insects, play a crucial role in osmoregulation and the selective reabsorption of water, ions, and solutes [8,9]. Our transcriptome and proteomic data revealed numerous genes and proteins associated with osmoregulation, organic solute transport, detoxification, and immunity (Figure 2D and Figure 3G). In some species, Malpighian tubules serve as secretory glands [8,9]. Brochosome synthesis is a prominent example of Malpighian tubule specialization [9]. Epithelial cells of the specialized glandular segment of the leafhopper Malpighian tubules, with a large spherical nucleus and an extensively developed rough endoplasmic reticulum, are typical of protein-secreting cells [3,4]. In this study, the transcriptomic analysis revealed that most of the highly expressed genes in the Malpighian tubules are involved in protein production. This suggests that *N. cincticeps* Malpighian tubules possess significant protein-synthesizing capabilities, which was further supported by the COG clustering analysis of the proteomic data, emphasizing energy supply, protein synthesis and modification-related functions (Figure 2D).

After molting, fully formed brochosomes are secreted through the hindgut and applied to the leafhopper’s integuments [2,5,31]. Although organic solvents have little effect on brochosomes [32], they can be used to wash brochosomes from leafhopper integuments. In our study, we collected brochosomes from the forewings of *N. cincticeps* using sonication and shaking methods with acetone as the solvent. Electron microscopy, gel electrophoresis, and LC–MS/MS results confirmed the efficient collection of brochosomes from leafhopper wings using both methods. The sonication method yielded higher brochosome isolation efficiency but introduced more impure proteins from the wings. On the other hand, the shaking method resulted in purer brochosomes (Figure 3).

During brochosome synthesis in Malpighian tubules, various other secretory proteins are produced and deposited onto the integuments along with brochosomes [18]. Interestingly, only a fraction of the proteins identified by LC–MS/MS included signal peptides, suggesting that many proteins lack secretory signals. These proteins likely originate from the liquid surrounding and filling mature brochosomes inside the secretory cells, subsequently released onto the integuments with brochosomes. This phenomenon may explain the abundance of proteins involved in osmoregulation, organic solute transport, detoxification, and immunity in our proteomic data (Figure 3G). Notably, cuticle proteins were the most abundant proteins in the proteome, with ultrasonic treatment showing exceptionally high peptide coverage of cuticle proteins (Table S6). This implies that many wing proteins were isolated when acetone was used as a solvent to separate brochosomes from wings. However, ultrasonication was more efficient in isolating brochosomes, albeit with higher protein contamination from the wings.

Previous studies have characterized the brochosome as a particle composed of a protein–lipid complex, with protein content ranging from 45–75% [2,16,17,18]. In the leafhopper species *G. fennahi* and *H. vitripennis*, the constituent proteins of the brochosome have been categorized as BSM and BSAP [18,19]. In our study, we identified 12 BSM proteins with conserved motifs and homology to *G. fennahi* BSM proteins (Figure 4, Figure 5 and Figure 6). These BSM proteins contain cysteine residues that may form intra- or interchain disulfide bonds, potentially explaining the tolerance and endurance of brochosomes in non-reducing buffers. The presence of protein disulfide-isomerase (PDI) in the endoplasmic reticulum (ER) is crucial for the formation and rearrangement of disulfide bonds during protein folding [33,34]. These observations suggest that BSAP proteins might play a role in the proper folding of BSM proteins and participate in the production of three-dimensional structures.

The specialization of the distal segment of the Malpighian tubule is a characteristic shared by the three major lineages of the infraorder Cicadomorpha: Cercopoidea (spittlebugs), Cicadoidea (cicadas), and Membracoidea (leafhoppers and treehoppers) [15]. However, in spittlebugs and cicadas, Malpighian tubules synthesize secretions only during the nymphal stage. For instance, the secretion produced by Malpighian tubules in cicada nymphs is considered fungicidal and fungistatic, protecting them in cryptobiotic micro-habitats in the soil [15]. Spittlebug nymphs have their integuments coated with froth synthetically produced by Malpighian tubules, which exhibit antifungal properties [13]. Previous studies have suggested the putative function of brochosomes in protecting leafhoppers from microbial infection by keeping the integument dry and inhibiting pathogenic fungal germination [1,2], although experimental verification is lacking. In our study, we found the attacin C conserved domain in *N. cincticeps* BSAP proteins (00009303-RA and 00009304-RA) (Figure 4). Attacin is a common antimicrobial peptide in insects, classified into basic and acidic types [35]. Attacin C, categorized as a basic attacin, has demonstrated excellent inhibitory activity against Gram-negative bacteria in *Drosophila melanogaster* [33]. This finding suggests that the antimicrobial peptides in BSAP might directly protect leafhoppers from pathogens.

All BSM proteins and some BSAP proteins identified in our study were exclusive to Cicadellidae, with no detectable homologs in other Hemiptera species, suggesting their encoding by a class of genes specific to Cicadellidae (Figure 5 and Figure 6). These species-specific genes, due to their lack of detectable similarity or homology to genes found in other species, are often referred to as orphan genes. Orphan genes exist in specific phylogenetic lineages and lack recognizable homologs in distantly related species, making it challenging to infer a clear signal of common descent (homology) [36,37,38]. While the origin and function of most orphan genes remain unknown, they are believed to play critical roles in species-specific developmental adaptations [39]. In the context of brochosome studies, the presence of species-specific secreted proteins encoded by orphan genes has been observed in other species. For example, in the robber fly Dasypogon diadema, a highly expressed spectrum-specific orphan gene encodes a unique venom protein [40]. In the Hessian fly (*Mayetiola destructor*), a secreted protein produced by an orphan gene plays a key role in the formation of the living feeding site gall [41]. Our findings, as well as previous research on *G. fennahi* [18], indicate that BSM and some BSAP proteins may be produced by orphan genes.

## 5. Conclusions

The study presented here focused on characterizing the protein composition of brochosomes in the rice green leafhopper *N. cincticeps* using transcriptomic and proteomic analyses. Utilizing proteomic techniques, the protein composition of brochosomes was examined. Brochosomes were obtained through ultrasound and shaking methods, leading to the identification of an impressive repertoire of 815 proteins within two samples. Moreover, a transcriptomic analysis highlighted the specific expression of 106 genes in the Malpighian tubules. Notably, this study delves even further, pinpointing 10 brochosome-associated proteins (BSAP) and 12 potential major structural proteins constituting the brochosome (BSM) of *N. cincticeps*. The origin of BSM proteins from Cicadellidae-specific orphan genes, housing conserved motifs, adds a layer of significance to their evolutionary context. Intriguingly, the BSAP components come forth as potential key players in ensuring proper BSM folding mechanisms and fortification against pathogens. These revelations underscore the intricate mechanisms of brochosome assembly and shed light on their vital functions in the realm of leafhopper biology.

## Data Availability

The mass spectrometry proteomics data were deposited in the ProteomeXchange Consortium via PRIDE [42] partner repository with the dataset identifier PXD039863. RNA-seq raw data are available at the NCBI Sequence Read Archive (SRA) database under BioProject IDs: PRJNA925901.

## Supplementary Materials

The following supporting information can be downloaded at: https://www.mdpi.com/article/10.3390/insects14100784/s1, Figure S1: COG functional classification of gene transcript levels TPM > 1 (A) and TPM > 100 (B) in Sg, Mg, Mt, and Rb of the leafhopper *N. cincticeps*. The columns are labeled as follows: A: RNA processing and modification; B: chromatin structure and dynamics; C, energy production and conversion; D, cell division and chromosome partitioning; E, amino acid transport and metabolism; F, nucleotide transport and metabolism; G, carbohydrate transport and metabolism; H, coenzyme metabolism; I, lipid transport and metabolism; J, translation, ribosomal structure and biogenesis; K, transcription; L, DNA replication, recombination and repair; M, cell wall/membrane biogenesis; N, cell motility; O, posttranslational modification, protein turnover, chaperones; P, inorganic ion transport and metabolism; Q, secondary metabolite biosynthesis, transport and catabolism; R, general function prediction only; S, function unknown; T, signal transduction mechanisms; U, intracellular trafficking and secretion; V, defense mechanisms; W, extracellular structures; Y, nuclear structure; and Z, cytoskeleton; Figure S2: Expression of Malpighian tube-specifically expressed genes validated by RT-qPCR in different tissues of *N. cincticeps*. Salivary glands, Sg; midgut, Mg; Malpighian tubules, Mt; and Residual body, Rb; Figure S3: Original unprocessed version of the SDS-PAGE gel included in Figure 3E of the main text; Figure S4: Sequences logos of the brochosome candidate proteins conserved motifs in Figure 4 of the main text; Table S1: Primers used in quantitative real-time PCR; Table S2: Summary of sequence assembly; Table S3: Transcriptome results; Table S4: Malpighian tube-specific expression genes; Table S5: Mateched unique peptides identified by LC-MSMS; Table S6: The protein identified by LC–MSMS.
