# Supplementary material for: Combined Transcriptome and Proteome Analysis of the Protein Composition of the Brochosomes of the Leafhopper Nephotettix cincticeps
Source: Insects. 2023 Sep 26;14(10):784. doi: 10.3390/insects14100784 (PMC10607721; doi:10.3390/insects14100784)
Supplement: Supplementary file 1 [file insects-14-00784-s001.zip › Supplementary Figure.pdf]

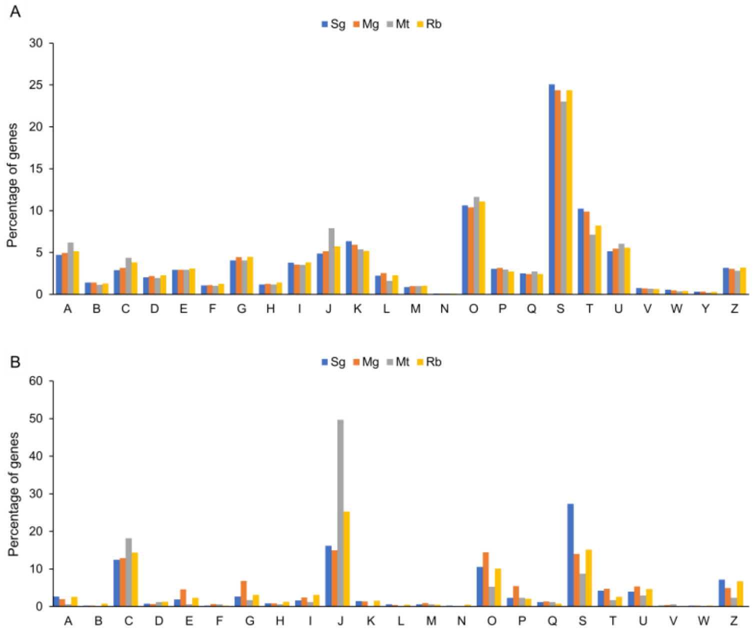

Supplementary Figure S1. COG functional classification of gene transcript levels TPM>1 (A) and TPM>100 (B) in Sg, Mg, Mt, and Rb of the leafhopper *N. cincticeps*. The columns are labelled as follows: A: RNA processing and modification; B: chromatin structure and dynamics; C, energy production and conversion; D, cell division and chromosome partitioning; E, amino acid transport and metabolism; F, nucleotide transport and metabolism; G, carbohydrate transport and metabolism; H, coenzyme metabolism; I, lipid transport and metabolism; J, translation, ribosomal structure and biogenesis; K, transcription; L, DNA replication, recombination and repair; M, cell wall/membrane biogenesis; N, cell motility; O, posttranslational modification, protein turnover, chaperones; P, inorganic ion transport and metabolism; Q, secondary metabolite biosynthesis, transport and catabolism; R, general function prediction only; S, function unknown; T, signal transduction mechanisms; U, intracellular trafficking and secretion; V, defense mechanisms; W, extracellular structures; Y, nuclear structure; and Z, cytoskeleton. Salivary glands, Sg; midgut, Mg; Malpighian tubules, Mt; and Residual body, Rb.

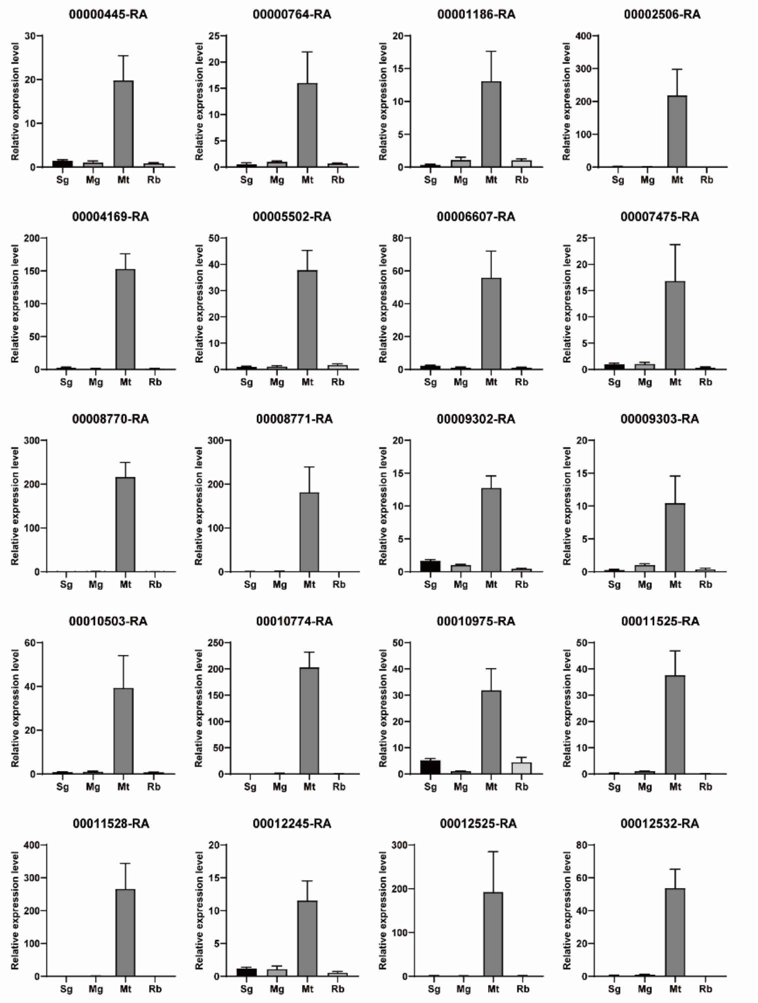

Supplementary Figure S2. Expression of malpighian tube-specifically expressed genes validated by RT-qPCR in different tissues of *N. cincticeps*. Salivary glands, Sg; midgut, Mg; Malpighian tubules, Mt; and Residual body, Rb.

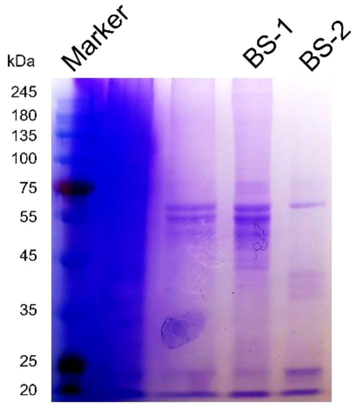

Supplementary Figure S3. Original unprocessed version of the SDS-PAGE gel included in Figure 3E of the main text.

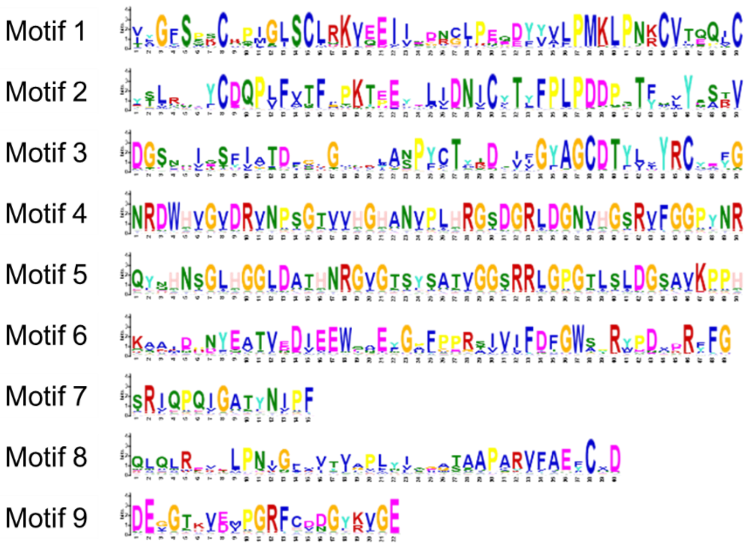

Supplementary Figure S4. Sequences logos of the brochosome candidate proteins conserved motifs in Figure 4 of the main text.
